# Supplementary figures and images for: Association between the metabolic score for insulin resistance and prostate cancer: a cross-sectional study in Xinjiang
Source: PeerJ. 2024 Jul 26;12:e17827. doi: 10.7717/peerj.17827 (PMC11285359; doi:10.7717/peerj.17827)

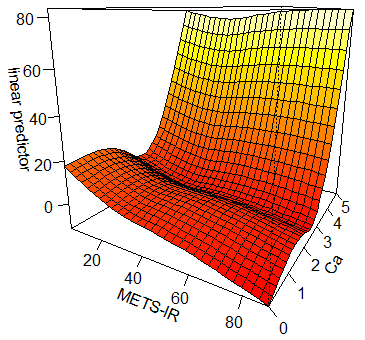

Supplement: Supplemental Information 1 [file peerj-12-17827-s001.png]

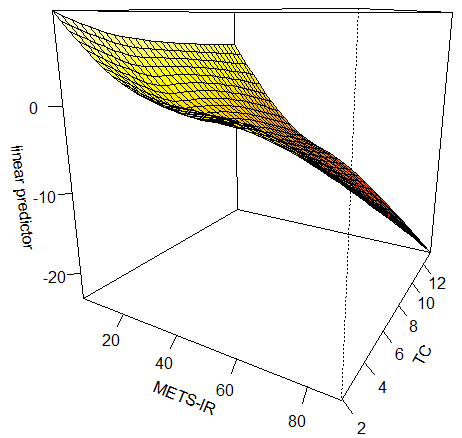

Supplement: Supplemental Information 2 [file peerj-12-17827-s002.png]

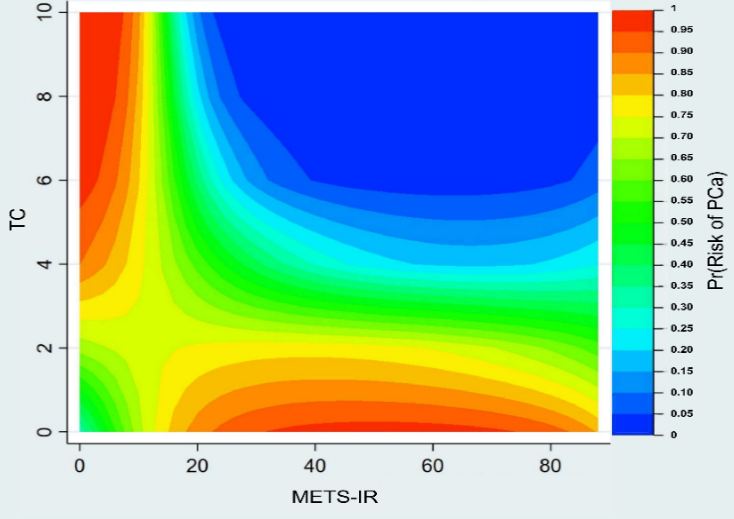

Supplement: Supplemental Information 3 [file peerj-12-17827-s003.png]
